# Concerning the Jequiriti Treatment of Granular Lids

**Published:** 1883-10

**Authors:** Lucien Howe


### Concerning the Jequiriti Treatment of Granular Lids.

By Lucien Howe, M. D.

Although the use of jequiriti or abrus precatorius, is comparatively new, enough has already been written concerning it to make its effects familiar even to those not especially interested in ophthalmological literature.

It will be remembered that it was recommended by DeWecker for the treatment of granular lids, especially those complicated with pannus. An infusion of the bean, when applied to the conjunctiva, was found to produce a severe inflammation of a diphtheritic nature, but when this subsided, it left the cornea clearer than before, and the granulations much smaller, if, indeed, they had not entirely disappeared. It had been known for a long time that the conjunctiva could be inoculated in a similar manner with gonorrhœal pus, and sometimes a favorable result obtained, but unfortunately the degree of inflammation could not be regulated, and frequently, instead of improving the original condition, the eye was thereby lost.

It was, therefore, a valuable discovery, when an agent was found to have all the advantages of that method of treatment, but free from most, if not all of its dangers. The usual tendency is always to over-estimate the efficacy of a new drug, but the results thus far obtained place one important fact beyond doubt, this is, that jequiriti is among the best agents at our command for treating certain aggravated forms of granular conjunctivitis, in selected cases even ranking above copper sulphate or silver nitrate. The clinical observations in support of this statement are already too numerous to warrant any account being given here of the ordinary experience with the drug. It may simply be remarked that I have thus far given the drug a fair trial in nine cases. Three of these were more improved by its use in about sixteen days than by application of sulphate of copper for nearly two months. In two, the improvement was also very decided, but it was necessary to induce the peculiar inflammation a second or third time. In two, it did absolutely no good. Finally, two others remain still under treatment, one of which is improving, and the other with no prospect of it. Or, to recapitulate: the results in three cases may be called very successful; in two, moderately good; in two, a complete failure, and in two, doubtful. I am aware that the data furnished by so few cases are not sufficient from which to draw any valuable conclusions as to the general plan of treatment, but the observations thus far made are certainly sufficient to warrant my calling attention to one or two facts regarding the use of the bean which writers have barely mentioned or entirely ignored.

The first of these relates to the freshness of the bean. Wecker laid some stress upon this point in one of his first articles, and attributed the failure of the treatment in the hands of others as perhaps due to their using old or dried beans. The importance of the observation is at least substantiated by my experience with the drug. The first beans which I used were obtained in June last, from Boston. An infusion of these, of two and one-half per cent., gave absolutely no results. One of five per cent. produced a moderate conjunctivitis, and finally a solution twice as strong was necessary to cause the effects usually described. Moreover, this occurred in only two eyes, out of the four on which it was then tried. Had I rested at that point and ventured to draw conclusions, they would have been rather unfavorable to the drug. Fortunately, however, I sent to another dealer, Mr. Fingerhut, of Fourth Avenue, New York, paying him rather a large price for a very small package of the beans. These were a brighter red than the first, but otherwise quite the same, to all appearances. On trying a two and one-half per cent. infusion on one of the eyes, which before was unaffected, I found the characteristic inflammation made its appearance before the third day, and an infusion twice as strong was sufficient to bring out the typical diphtheritic deposit and swelling of lids and face. This was producced in each of the four cases already mentioned, and still a fifth, subjected to the treatment for the first time. Of these five, two then complained, in addition, of very decided vertigo, and one was not only confined to her bed in the hospital for two days, but also had hallucinations similar to those which occasionally accompany the use of an opiate. The difference in the action of these two specimens of the drug is sufficient to warrant some care in its selection.

A second point to which I would call attention is the diminishing effect of the same infusion, when used for a length of time. I noticed this particularly when experimenting with jequiriti on the eyes of rabbits. For example, a two and one-half or five per cent. solution, even of the stale beans, would set up a conjunctivitis (but not of diphtheritic nature), which would reach a maximum degree about the end of the second or beginning of the third day, and gradually subside, in spite of the continuance of the applications, although it would not entirely disappear. If a fresh infusion of the same beans was then used, more acute symptoms would again supervene. The same variations I have witnessed in the human subject. I am frank to say that usually, when the degree of inflammation has been very severe, I dared not continue the jequiriti simply to determine how much the eye would still tolerate. In the case of an old man, however, with a very thick pannus, and granulations of several years' standing, I used a five per cent. solution, till the diphtheritic deposit, pain and swelling, was produced; but these symptoms gradually subsided, and in the course of two weeks almost entirely disappeared, although applications were continued three times a day.

The natural inference from this is, that the infusion deteriorates quite rapidly, and although I am told that at one large Infirmary in this country, only fresh infusions are used, still I do not think this fact has received the attention in the literature, which its importance demands.*

The third point to which I would call attention is the effect of jequiriti infusion upon the cornea. DeWecker insists that this structure is not endangered by its use, and I am only aware of one writer who has allowed this statement to pass unchallenged. My experience, however, would lead me to think that the cornea is by no means free from danger by this method of treatment. In two cases I have seen an ulcer form even when the degree of inflammation otherwise was not in a very great degree. As soon as the applications were withheld, and atropine used, the healing of the ulcers soon began, but without doubt they were due to the jequiriti treatment.

Although such ulcerations may be small and superficial, and although they may be rare, still, one such observation is sufficient

* Annales D'Oculistique Mai-Juin, 1883, page 218.

to make the surgeon cautious in receiving the statement that "the cornea runs no risk during the development of jequiriti opthalmia."

These three facts concerning the use of the drug seem to be of so much importance as to be worthy of special attention by those who avail themselves of its undoubted advantages.

